# Rtn1a-Mediated Endoplasmic Reticulum Stress in Podocyte Injury and Diabetic Nephropathy

**DOI:** 10.1038/s41598-017-00305-6

**Published:** 2017-03-23

**Authors:** Ying Fan, Jing Zhang, Wenzhen Xiao, Kyung Lee, Zhengzhe Li, Jiejun Wen, Li He, Dingkun Gui, Rui Xue, Guihua Jian, Xiaohua Sheng, John Cijiang He, Niansong Wang

**Affiliations:** 1 Department of Nephrology, Shanghai Jiao Tong University Affiliated Sixth People’s Hospital, Shanghai, China; 2 Department of Medicine, Division of Nephrology, Icahn School of Medicine at Mount Sinai, NY, United States; 3 Renal Section, James J Peter Veterans Administration Medical Center, Bronx, NY United States

## Abstract

We previously reported a critical role of reticulon (RTN) 1A in mediating endoplasmic reticulum (ER) stress in kidney tubular cells and the expression of RTN1A correlates with the renal function and the severity of kidney injury in patients with diabetic nephropathy (DN). Here, we determined the roles of RTN1A and ER stress in podocyte injury and DN. We used db/db mice with early unilateral nephrectomy (Unx) as a murine model of progressive DN and treated mice with tauroursodeoxycholic acid (TUDCA), a specific inhibitor of ER stress. We found increased expression of RTN1A and ER stress markers in the kidney of db/db-Unx mice. Treatment of TUDCA not only attenuated proteinuria and kidney histological changes, but also ameliorated podocyte and glomeruli injury in diabetic mice, which were associated with reduction of RTN1A and ER stress marker expression in the podocytes of TUDCA-treated mice. *In vitro*, we showed RTN1A mediates albumin-induced ER stress and apoptosis in human podocytes. A positive feedback loop between RTN1A and CHOP was found leading to an enhanced ER stress in podocytes. Our data suggest that ER stress plays a major role in podocyte injury in DN and RTN1A might be a key regulator of ER stress in podocytes.

## Introduction

Diabetic nephropathy (DN) remains the most common cause of chronic kidney disease (CKD) and end stage renal disease (ESRD), accounting for nearly 50% of new cases of renal failure in the United States in 2008^1^. Clinically, DN is defined by the appearance of microalbuminuria at the early stage that progresses toward ESRD over time. Histologically, DN is characterized by glomerular basement membrane (GBM) thickening, mesangial expansion, podocyte and tubular cell injury (apoptosis) at the early stage and diffuse/nodular glomerulosclerosis and tubulointerstitial fibrosis at the later stage. Many studies have shown that podocyte dysfunction plays a crucial role in causing the defective glomerular filtrations as well as the onset of proteinuria^2, 3^. Since podocytes are highly differentiated cells with limited capability to undergo cell division in the adult, loss of podocytes due to either detachment or apoptosis leads to reduction of podocyte numbers. Reduction of podocyte number together with glomerular hypertrophy contributes to the low podocyte density in diabetic kidney, which is a strongest predictor of progression of DN and correlates directly with the magnitude of proteinuria^4^.

Several lines of evidence suggest that endoplasmic reticulum (ER) stress plays a major role in the development and progression of DN^5–7^. High glucose concentrations induce ER stress and apoptosis of kidney cells^8^. Diabetic CHOP knockout mice seemed to be protected from DN, since they developed less proteinuria than wild type diabetic mice^9^. In addition, renal expression of ER stress markers in patients with established DN is higher than patients with mild DN, suggesting a heightened ER stress response in the kidney of DN patients^8^. Elevated urinary protein excretion in humans is also known to be associated with tubular injury and ER stress^8, 10, 11^. In humans and mice with nephrotic syndrome, ER stress markers are elevated in tubular epithelial cells^12, 13^. Podocytes are likely susceptible to ER stress due to its large capacity of ER and high level of anabolic or catabolic activities^14^. Several studies suggest an association of ER stress with podocyte injury^6, 15^. However, the mechanism of ER stress mediated podocyte injury in DN remains largely unknown.

In the previous study^16^, we identified a critical role of endoplasmic reticulum (ER)-associated protein reticulon-1A (RTN1A) in regulation of ER stress in kidney tubular cells. Knockdown of RTN1A expression *in vivo* attenuates ER stress and renal fibrosis^17^. Further, increased expression of RTN1A strongly correlates with the progression of DN and the severity of kidney injury. However, whether RTN1A and ER stress contributed to podocyte injury in DN had not been studied in detail.

Therefore in the current study, we examined whether inhibition of ER stress by Tauroursodeoxycholic acid (TUDCA), a well-known ER stress inhibitor, attenuated podocyte injury and diabetic kidney disease through regulation of RTN1A and ER stress markers in a murine model of progressive DN. In addition, we examined the role RTN1A in regulation of albumin-induced ER stress in cultured podocytes. Our studies provide us a better understanding of ER stress in inducing podocyte injury in DN.

## Results

### Accelerated kidney injury in db/db-Unx mice was attenuated by TUDCA treatment

**Figure 1:**
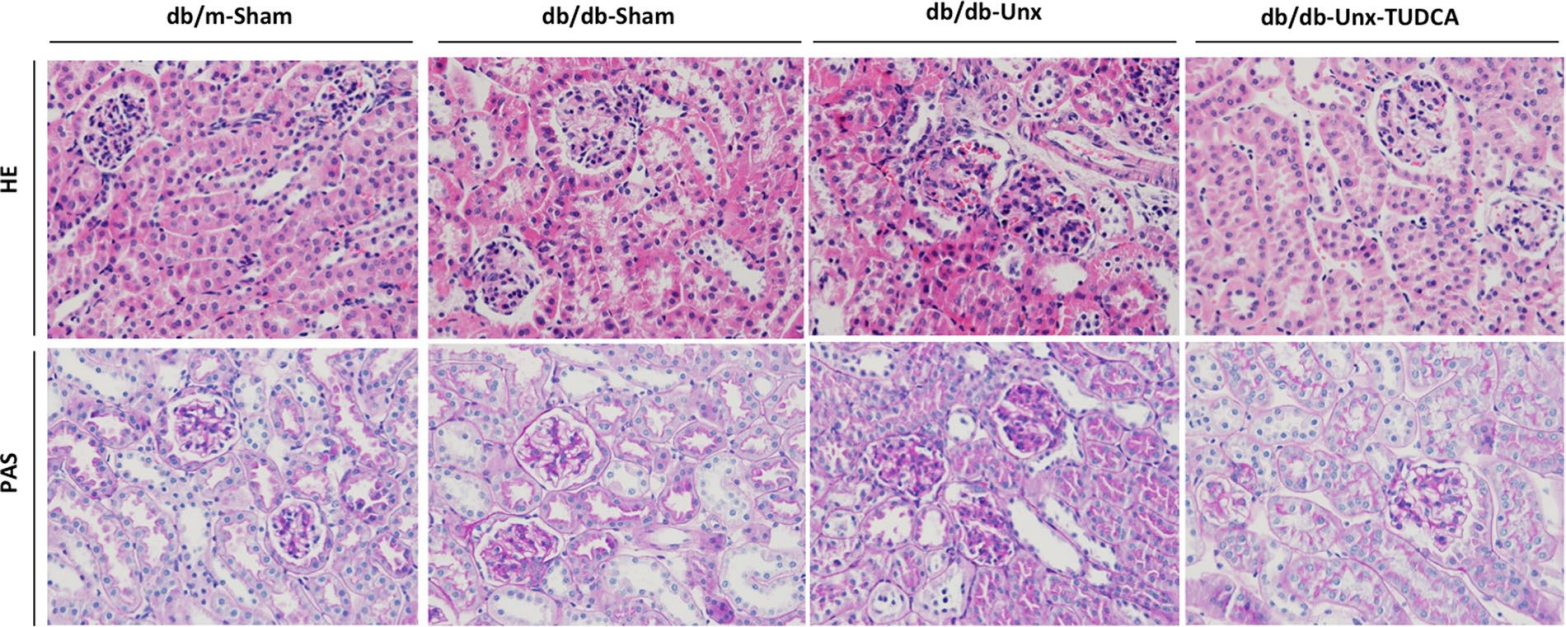
Histological changes of kidney were attenuated in db/db-Unx mice treated with TUDCA. Haematoxyline eosin and periodic acid-schiff staining of kidney sections were compared between different groups of mice (Original magnification ×400).

In this study, male db/db mice and their db/m littermates on C57BLKS/J background underwent Unx or sham operation at the age of 8 weeks. All db/db mice had increased body weight and blood glucose levels compared with db/m mice at 16 weeks of age. db/db mice that underwent Unx (db/db-Unx) had increased serum creatinine level, increased urine albumin/creatinine ratio (UACR), and more severe kidney histological changes at 16 weeks of age compared with age matched db/db-sham mice as well as db/db-sham mice at 20 weeks of age (Supplementary Table 1, Supplementary Fig. 1). Hence, early Unx of male db/db mice accelerated kidney injury and could be used as a murine model of progressive diabetic nephropathy and we used this mouse model to test whether inhibition of ER stress attenuates kidney injury in DN. Two weeks after Unx or sham operation, we intraperitoneally injected db/db mice with TUDCA (250 mg/kg), an ER stress reducing chemical chaperone, or equal volumes of saline twice daily for 6 weeks. The db/db-Unx mice treated with TUDCA had greatly reduced proteinuria, improved renal function and attenuated kidney injury (Table 1, Fig. 1), suggesting that inhibition of ER stress by TUDCA, a pharmacological inhibitor of ER stress, attenuated proteinuria and kidney injury in diabetic mice.

**Table 1:** db/db-Unx mice developed pronounced albuminuria at 16 week of age and were alleviated by TUDCA treatment.

	db/m-Sham	db/db-Sham	db/db-Unx	db/db-Unx-TUDCA
Body Weight (BW,g)	26.27 ± 1.69	54.53 ± 4.70*	46.43 ± 4.02*	47.05 ± 6.12*
Kidney Weight (KW,mg)	156.8 ± 5.75	239.0 ± 20.78*	369.0 ± 10.86*^#^	287.6 ± 43.10*^ϕ^
KW/BW ratio (mg/g)	5.99 ± 0.50	4.43 ± 0.81	7.98 ± 0.56*^#^	6.15 ± 1.03^ϕ^
Blood glucose (mM)	8.0 ± 1.2	27.6 ± 1.9*	27.3 ± 4.2*	17.9 ± 3.1*^ϕ^
sCr (mg/dl)	0.25 ± 0.09	0.43 ± 0.11	0.98 ± 0.10*^#^	0.50 ± 0.17*^ϕ^
UACR (ug/mg)	54.8 ± 12.6	1074.1 ± 171.5*	2011.1 ± 130.3*^#ϕ^	1473.9 ± 181.9*^#ϕ^
UAE (ug/12 h)	13.4 ± 1.4	193.1 ± 30.0*	367.3 ± 25.9*^#^	292.4 ± 9.2*^#ϕ^

Abbreviations: Sham, sham operation; Unx, uninephrectomy; BW, body weight; KW, kidney weight; sCr, serum creatine; UACR, urine albumin/creatinine ratio; UAE, urine albumin excretion.

Clinical and laboratory parameters of db/m, db/db mice and db/db/Unx mice treated with or without TUDCA. Values are expressed as means ± SEM. *P < 0.05 compared to db/m-shame groups, ^#^P < 0.05 vs db/db-sham mice, ^ϕ^P < 0.05 vs db/db-Unx mice, n = 8.

### Increased expression of RTN1A and ER stress markers in kidney of db/db-Unx mice was attenuated by TUDCA treatment

**Figure 2:**
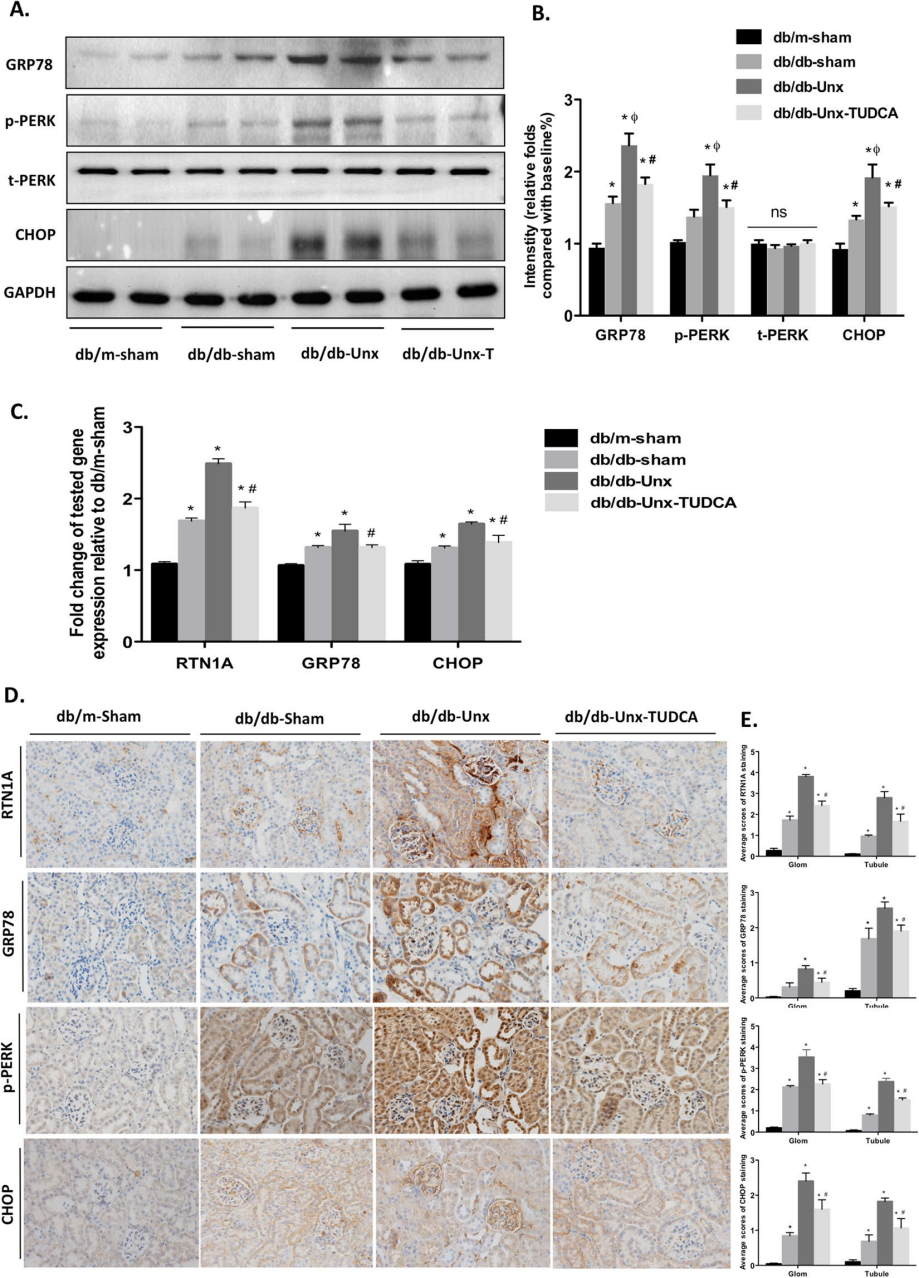
Expression of RTN1A and ER stress markers was increased in the kidney of db/db-Unx mice, which was suppressed by TUDCA treatment. (**A**) The kidney tissue lysates were subjected to immunoblot analysis with specific antibodies against t-PERK, p-PERK, GRP78 and CHOP. Full-length blots/gels are presented in Supplementary Figure S2. (**B**) The densitometry analyses of western blots are shown. (**C**) Real-time PCR showed an increase in mRNA expression of RTN1A, GRP78 and CHOP in kidney tissues. Results are means ± SEM. *P < 0.05 compared with db/db-shame group, ^#^P < 0.05 compared with db/db-Unx group, n = 5. (**D**) Immunohistochemistry staining for RTN1A and ER stress markers in kidneys and the representative pictures are shown (Original magnification x400). (**E**) Semi-quantitative data of RTN1A, p-PERK, GRP78 and CHOP staining in different groups of mice. Data are expressed as means ± SEM. *P < 0.05 versus db/m-Sham group; ^#^P < 0.05 versus db/db-Unx group; ^ϕ^P < 0.05, versus db/db-Sham group. n = 5.

To determine whether the protective effect of TUDCA in db/db mice was via inhibiting ER stress pathways, we examined the renal expression of ER stress markers in the mice. We found that expression of RTN1a and ER stress markers (GRP78, p-PERK and CHOP) was significantly increased in the kidneys of db/db-Unx mice, as measured by either western blot or real-time PCR (Fig. 2A–C). This was also confirmed by immunostaining of RTN1A and other ER stress markers (Fig. 2D and E). In db/db-Unx mice treated with TUDCA, expression of RTN1A as well as GRP78, p-PERK and CHOP were suppressed at both protein and mRNA level, indicating a protective role of TUDCA in DN via inhibiting RTN1A and ER stress in kidney cells (Fig. 2A–E). Immunostaining of RTN1A showed more pronounced expression in the glomerular area than tubular compartment of db/db mice, which is consistent with the observation that diabetic mice have more injury in the glomeruli than in tubules. These data suggest a role of RTN1A in ER stress-mediated glomeruli injury in DN.

### TUDCA protected glomeruli from diabetes-induced podocyte injury in db/db mice

**Figure 3:**
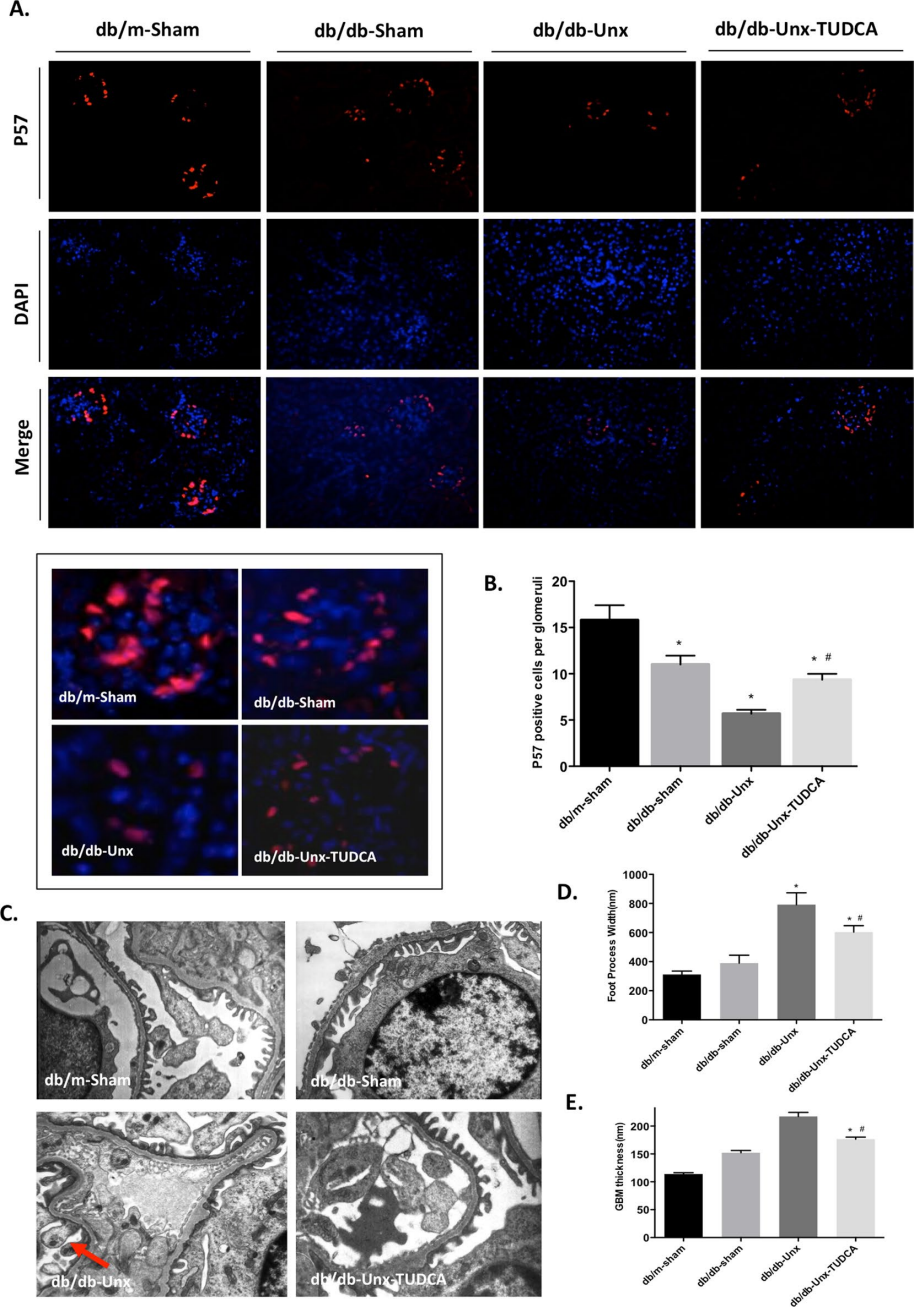
TUDCA protected glomeruli from diabetes induced podocyte injury in db/db-Unx mice. (**A**) Immunofluorescence stainning of p57 with DAPI for nuclear counterstaining (Original magnification ×200). Representative pictures on the nuclear staining of the podocyte marker P57 are shown on the right panel (Original magnification ×400). (**B**) Quantification of P57 positive podocytes per glomerular cross section in db/m and db/db mice. Values are expressed as means ± SEM. *P < 0.05 compared with db/m-sham mice, ^#^p < 0.05 compared with db/db-Unx mice, n = 5. (**C**) Electron microscopic photographs to illustrate the morphological changes in the podocyte foot process and GBM thickness in different groups of mice. Quantification shows (**D**) FPs and (**E**) GBM thickness in the glomeruli of mice from each group. Results are from measurements performed in 3–4 glomeruli from 3 mice per group. *P < 0.05 compared with db/m-sham mice, ^#^P < 0.05, compared with db/db-Unx mice, n = 3.

We next performed immunofluorescence staining of P57, which is expressed only in podocytes in glomeruli, to examine the podocyte number in glomeruli of diabetic mice. db/db-Unx mice showed a significant reduction in the podocytes number per glomerulus compared with db/db-sham mice and db/m-sham mice. The podocyte number was partially restored in TUDCA-treated diabetic mice compared to vehicle-treated db/db mice (Fig. 3A and B). By electron microscopy we found that db/db-Unx mice exhibited diffuse podocyte effacement and foot process (FP) widening (Fig. 3C). Quantification showed significantly broader FPs and slightly thickened glomerular basement membrane (GBM) in the glomeruli of db/db-Unx mice (Fig. 3D and E) compared to sham operated db/db or db/m mice. Ultrastructural study also detected swelling and vacuolar changes of podocyte in db/db-Unx mice (Arrow). As expected, we found that podocytes loss was partially restored and the morphometric changes were also ameliorated in TUDCA treated diabetic mice, suggesting that treatment with TUDCA could ameliorate glomeruli dysfunction and podocyte injury via suppression of ER stress in db/db-Unx mice.

### TUDCA protects glomeruli against diabetes-induced apoptosis in db/db-Unx mice

**Figure 4:**
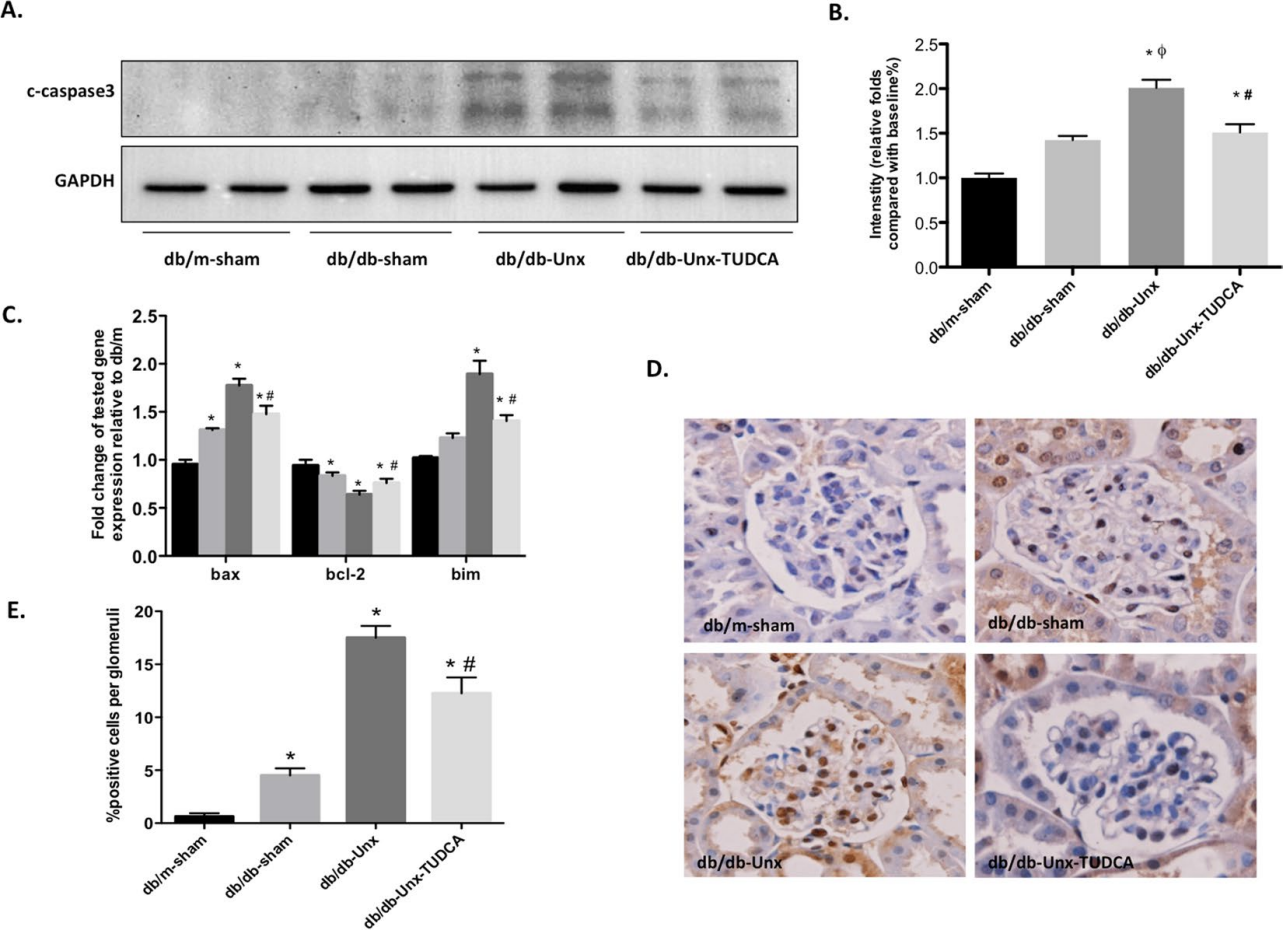
TUDCA protected glomeruli against diabetes induced cell apoptosis in db/db-Unx mice. (**A**) TUDCA reduced apoptosis in glomeruli of diabetic mice by Western blot analysis of caspase-3. Full-length blots/gels are presented in Supplementary Figure S2. (**B**) The densitometry analyses are performed for western blots. (**C**) Expression of pro-apoptotic genes (bax, bcl-2, and bim) were measured by Real-time PCR. (**D**,**E**) TUNEL staining and quantification were performed to determine the apoptosis of glomerular cells in each group of mice. Values are expressed as means ± SEM. *P < 0.05 versus db/m-Sham group; ^#^P < 0.05 versus db/db-Unx group; ^ϕ^P < 0.05, versus db/db-Sham group. n = 5.

It has been reported that prolonged ER stress leads to apoptosis of kidney cells in murine models of kidney diseases^16^. Our data showed that TUDCA reduced apoptosis in glomeruli of diabetic mice as accessed by western blot of cleaved caspase-3 (Fig. 4A and B) and real-time PCR analysis of apoptotic markers (bax, bcl-2, and bim) (Fig. 4C). In addition, we performed TUNEL staining to measure the apoptotic cells of glomeruli. TUNEL-positive cells were increased in the glomeruli of db/db-Unx mice, which were reduced in TUDCA-treated db/db-Unx mice, as compared to vehicle-treated db/db-sham or db/db-Unx mice (Fig. 4D and E). Taken together, these data indicate TUDCA protects glomeruli against diabetes-induced cell apoptosis in db/db-Unx mice. Although the exact identify of these apoptotic glomerular cells is not clear, podocyte is likely one of them.

### RTN1A expression in podocytes was highly associated with ER stress and podocyte injury in the glomeruli of diabetic mice

**Figure 5:**
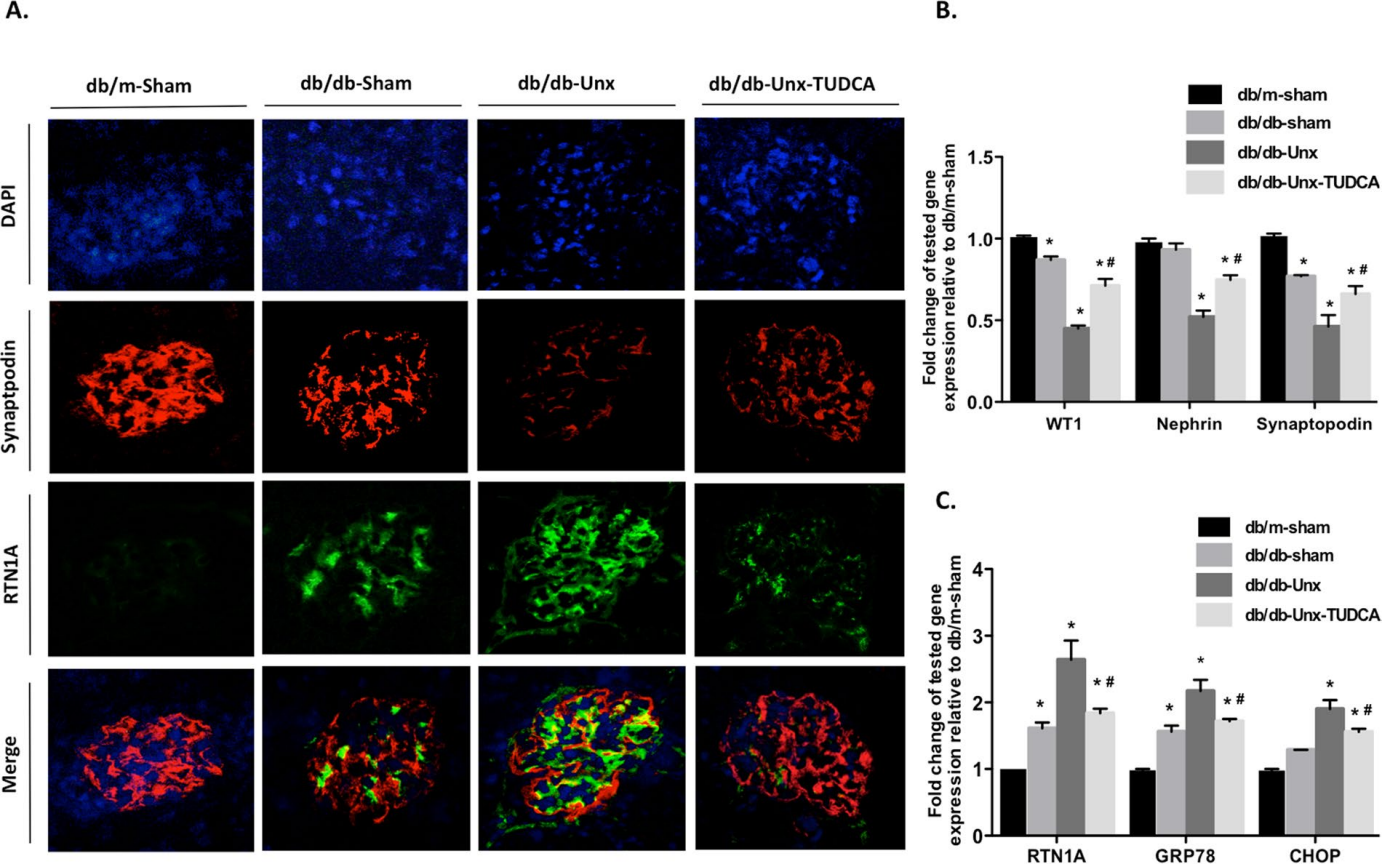
RTN1A expression in podocytes was highly associated with ER stress and podocyte injury in the glomeruli of diabetic mice. (**A**) Immunofluorescent staining (magnification ×400) illustrates RTN1A (green) and synaptopodin (red) co-staining in kidney sections. (**B**) Gene expressien of podocyte markers by real-time PCR analysis of glomeruli from mice in different groups (**C**) Gene expression of RTN1A and ER stress markers by real-time PCR analysis of glomeruli from mice in different groups. Values are expressed as means ± SEM. *P < 0.05 compared with db/m-sham mice, ^#^P < 0.05, compared with db/db-Unx mice, n = 5.

To determine the relationship between RTN1A and podocyte injury, we performed co-immunofluorescence staining for RTN1A and podocyte marker synaptopodin in kidney sections from these mice. RTN1A was co-localized with synaptopodin, indicating the expression of RTN1A in podocytes (Fig. 5A). The expression of RTN1A was significantly increased and while expression of synaptopodin was decreased in the podocytes of Unx-db/db mice and these were reversed after TUDCA treatment (Fig. 5A). By real-time PCR measurement, we showed that down-regulation of podocyte markers and up-regulation of RTN1A and respective ER stress markers in isolated glomeruli of db/db-Unx mice were also suppressed after TUDCA treatment (Fig. 5B and C). Taken together, these data indicate a significant association of RTNA1 expression with ER stress and podocyte injury in the glomeruli of diabetic mice.

### TUDCA ameliorates RTN1A-induced ER stress and apoptosis in human podocytes

**Figure 6:**
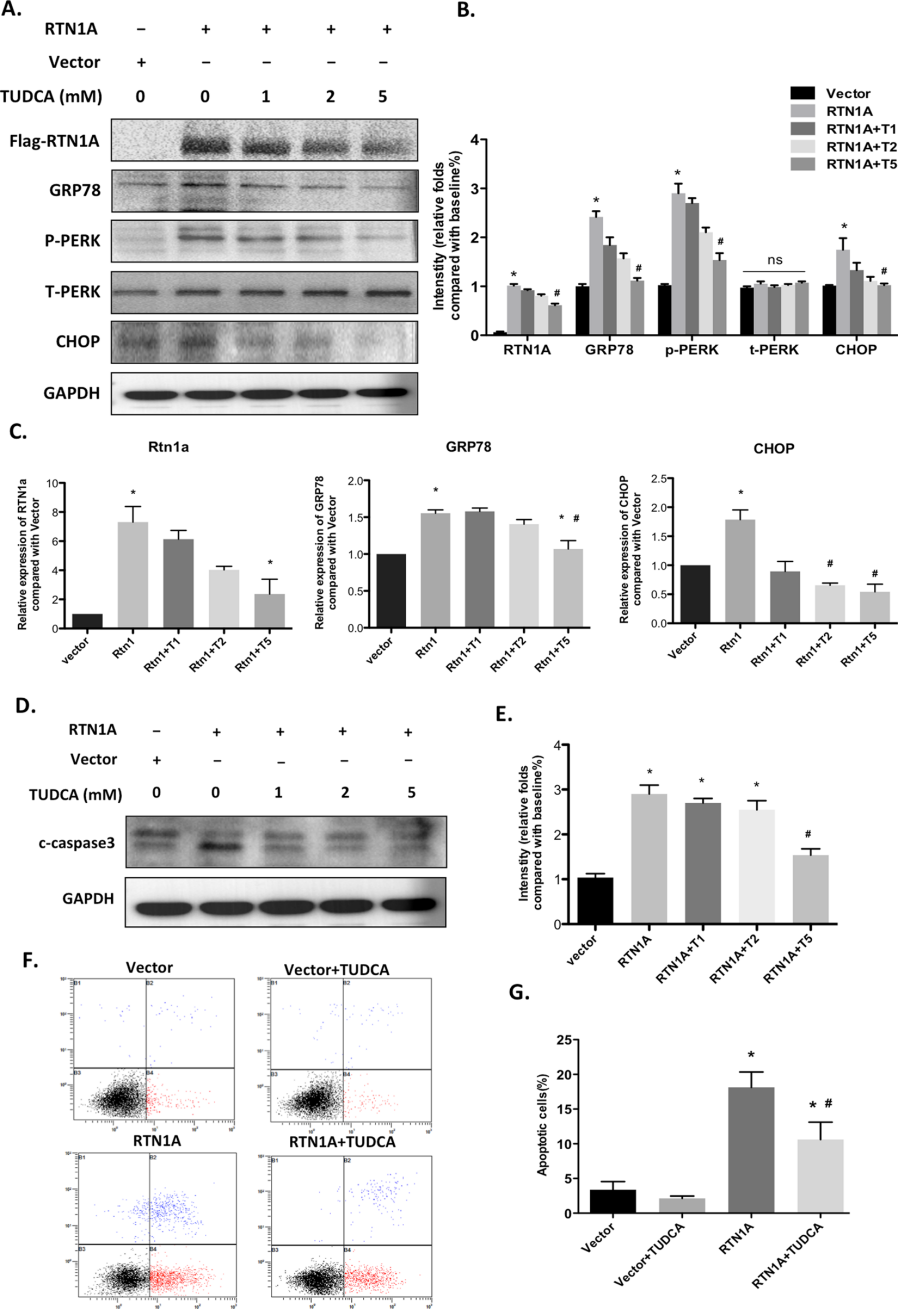
RTN1A-induced ER stress and apoptosis in podocytes was attenuated by TUDCA treatment. Human podocytes were transfected with RTN1A with or without TUDCA treatment for 48 h. (**A**) Western blot analysis with specific antibodies against t-PERK, p-PERK, GRP78 and CHOP. Full-length blots/gels are presented in Supplementary Figure S2. (**B**) The densitometry analyses of western blots for ER stress markers are shown. (**C**) mRNA levels of ER stress markers (RTN1A, GRP78 and CHOP) were measured by Real time-PCR analysis (**D**) Cleaved caspase3 was measured by western blot. Full-length blots/gels are presented in Supplementary Figure S2. (**E**) The densitometry analyses of western blots for c-caspase3 are shown. (**F**) Flow cytometry after double labeling with Annexin V and propidium iodide. (**G**) Quantification of apoptotic cells. Values are expressed as means ± SEM. *p < 0.05 compared to vector cells, ^#^P < 0.05 compared to RTN1A transfected cells, n = 3.

To further confirm the role of RTN1A in regulation of ER stress in podocytes, we performed *in vitro* study in cultured human podocytes. We found that overexpression of RTN1A in podocytes directly induced ER stress, as reflected by increased expression of RTN1A, PERK phosphorylation and GRP78 and CHOP expression. However, RTN1A-induced expression of ER stress markers was significantly attenuated by TUDCA treatment (Fig. 6A–C). Overexpression of RTN1A also induced podocytes apoptosis, which was also alleviated by TUDCA treatment, as assessed by the measurement of cleaved caspase-3 (Fig. 6D and E) and Annexin V labeling (Fig. 6F and G). These results confirm a role of RTN1A in ER stress in podocytes, since treatment with TUDCA led to a blockade of RTN1A mediated ER stress and apoptosis in podocytes.

### RTN1A mediates albumin-induced ER stress and apoptosis in human podocytes

**Figure 7:**
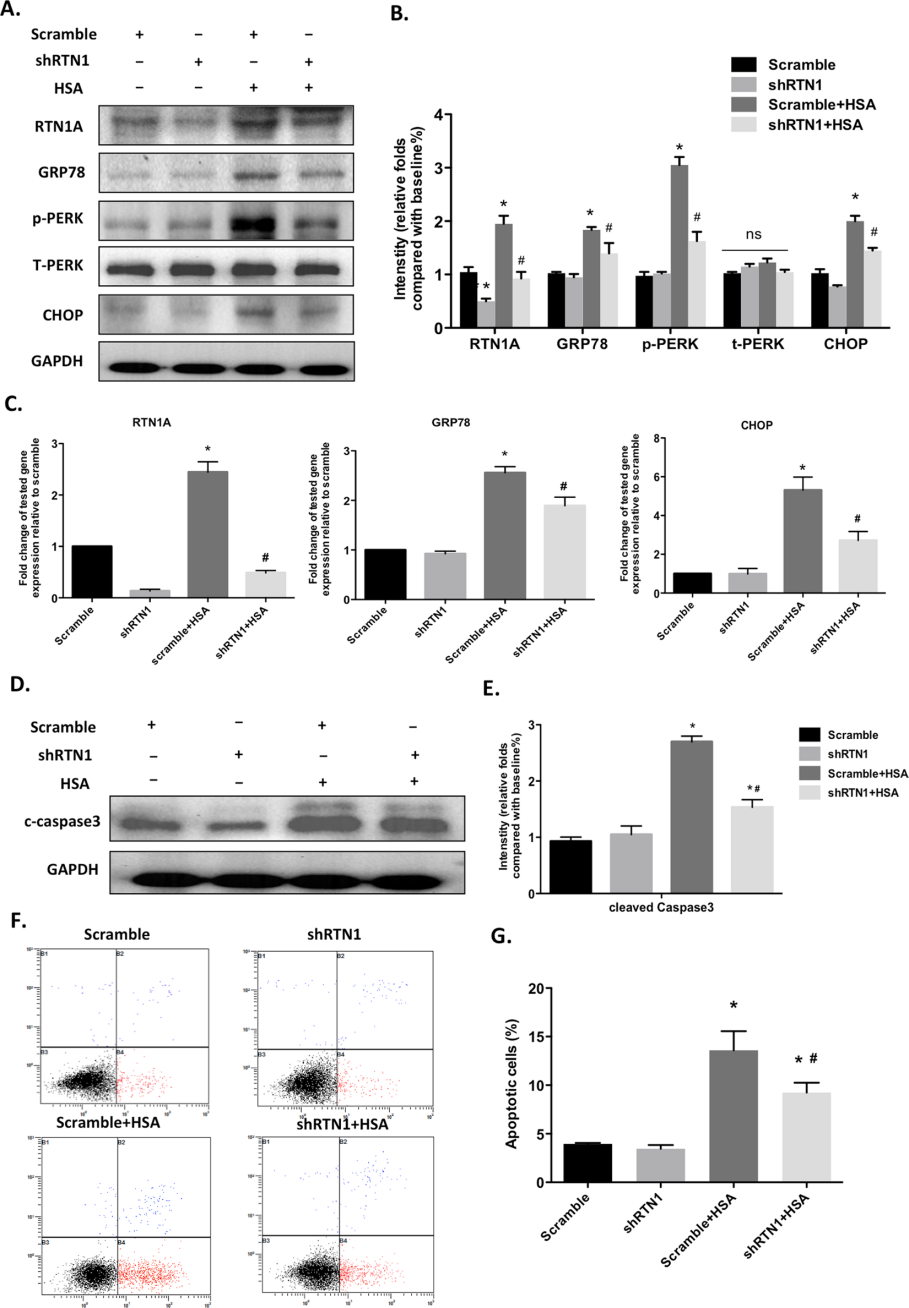
Knockdown of RTN1A attenuated albumin-induced ER stress and apoptosis in human podocytes. Human podocytes were transfected with shRNA-RTN1A or scramble shRNA followed by treatment with or without human serum albumin (HSA) for 48 h. (**A**) The proteins expression of RTN1A and ER stress markers (PERK, GRP78 and CHOP) was assessed by Western blot. Full-length blots/gels are presented in Supplementary Figure S2. (**B**) The densitometry analyses of western blots for ER stress markers are shown. (**C**) Real time PCR analyses were performed for genes of RTN1A and ER stress markers (GRP78 and CHOP). (**D**) The proteins expression of cleaved caspase-3 was detected by Western blot. Full-length blots/gels are presented in Supplementary Figure S2. (**E**) The densitometry analyses of western blots are shown. (**F**) Cell apoptosis was assessed by flow cytometer with Annexin V-FITC and PI. (**G**) Quantification of apoptotic cells. Values are expressed as means ± SEM. *P < 0.05 compared to scramble; ^#^P < 0.05 compared to Scramble+HSA, n = 3.

To further validate the role of RTN1A in mediating ER stress and podocyte apoptosis, we determined whether RTN1A also mediated albumin-induced ER stress and apoptosis in podocytes. We found that knockdown of RTN1A attenuated HSA-induced ER stress in human podocytes, as reflected by decreased phosphorylation of PERK and expression of GRP78 and CHOP in human podocytes (Fig. 7A–C), indicating that podocyte ER stress was activated in response to HSA stimulation and was alleviated by knockdown of RTN1A. In addition, we investigated the role of RTN1A in albumin-induced podocyte apoptosis and found that knockdown of RTN1A expression protected podocytes from apoptosis as assessed by western blot of cleaved caspase-3 (Fig. 7D and E) and Annexin V flow cytometry (Fig. 7F and G). These data indicate that RTN1A also contributes to protein overloading induced ER stress and plays a critical role in podocyte injury and apoptosis.

### RTN1A expression was regulated by downstream transcriptional factor CHOP

**Figure 8:**
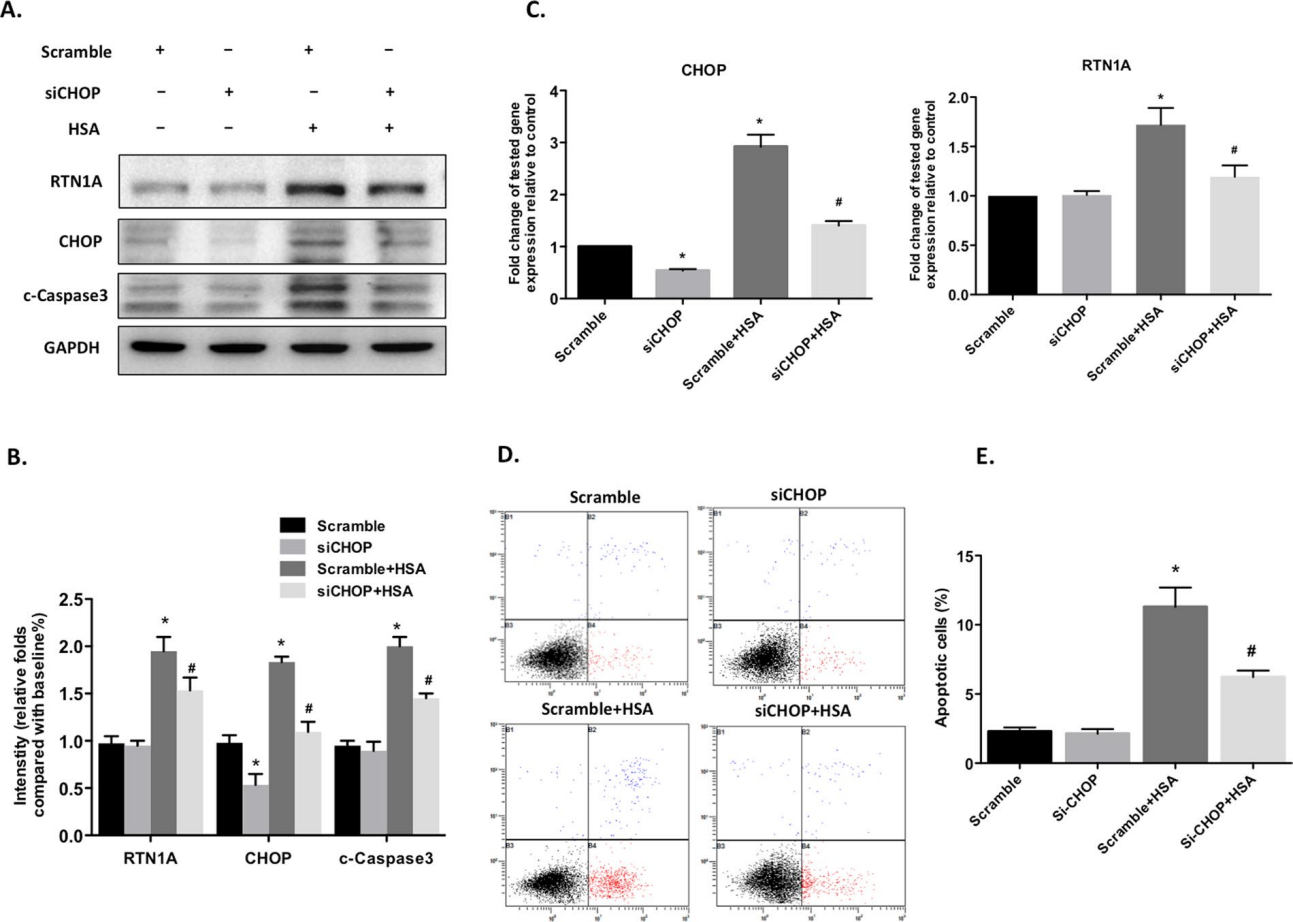
Knockdown of CHOP suppressed RTN1 expression, ER stress and apoptosis in albumin-treated human podocytes. Human podocytes were transfected with CHOP siRNA or scramble shRNA. 48 hours after transfection, cells were then treated with or without human serum albumin (HSA) for another 48 h. (**A**) The proteins expression of RTN1A, CHOP and cleaved caspase3 were assessed by western blot. Full-length blots/gels are presented in Supplementary Figure S2. (**B**) The densitometry analyses western blots are shown. (**C**) Real-time PCR analyses were performed for gene expression of RTN1A and CHOP. (**D**) Flow cytometry was performed to analyze podocyte apoptosis after double labeling with Annexin V and propidium iodide. (**E**) Quantification of apoptotic cells. Values are expressed as means ± SEM. *P < 0.05 compared to scramble group. ^#^P < 0.05 compared to scramble+HSA group, n = 3.

Finally, to understand how RTN1A contributes to podocyte ER stress and apoptosis, we examined the interaction between RTN1A and CHOP, a key ER stress transcriptional factor leading to the activation of apoptosis. We knocked down CHOP with siRNA in human podocytes, then stimulated with HSA for 48 h. Our results showed that knockdown of CHOP significantly suppressed RTN1A expression, indicating a positive feedback loop between CHOP and RTN1A. Knockdown of CHOP also suppressed HSA-induced ER stress in human podocytes as determined by western blot and real-time PCR (Fig. 8A–C). In addition, CHOP depletion also protected podocytes from HSA-induced apoptosis assessed by flow cytometry analysis using Annexin V labeling (Fig. 8D and E). Taken together, our data suggest that CHOP has a positive feedback to RTN1A.

## Discussion

Regardless of the current treatment for diabetic nephropathy (DN), nearly half of these patients inevitably progress to end stage of renal disease^18^. Therefore, it is extremely urgent to develop more effective therapy for DN. A large body of evidence suggests that glomeruli dysfunction and podocytes injury are key events in the pathogenesis of DN^19, 20^. Reduction of podocyte number due to apoptosis and detachment correlates with the amount of proteinuria and rate of progression in DN^21^. Thus, it is important to understand the mechanism of podocyte injury in order to develop specific treatment against podocyte loss in DN.

Although several groups reported a role of ER stress in DN, most studies have been done in animal models with STZ-induced diabetes, which have mild and early diabetic kidney injury^8, 22^. To better understand the role of ER stress in the progression of DN, here we used the db/db mice with Unx to better mimic progressive DN in human. While C57BLKS/J db/db mice at 20–24 weeks of age usually exhibit more profound diabetic kidney injury than STZ-induced diabetic mice, they nevertheless do not display significant histologic changes that are typical in human DN kidneys. Consistent with a previous report^23^, we found the degree of albuminuria in C57BLKS/J db/db mice did not increase consistently after 16 weeks of age. To hasten the development of DN, we performed Unx in db/db mice at 6 weeks of age, resulting in more severe histopathological changes, significantly increased UACR and serum creatinine level as compared to sham-operated db/db group. Therefore, we believe that db/db mouse with Unx is a better model to study the progression of DN than db/db mouse. Expression of ER stress markers in the kidney was also much higher in db/db-Unx than db/db mice, suggesting db/db-Unx is also a good model to study the role of ER stress in the progression of DN. Here, we have shown that inhibition of ER stress improved diabetic kidney injury in db/db-Unx mice.

Here, we reported a novel ER stress marker, RTN1A, which was highly expressed in diabetic kidney and suppressed by TUDCA, an inhibitor of ER stress. Our previous study suggests that RTN1A is a stimulator of ER stress in multiple kidney disease such as HIVAN and DN and plays a critical role in kidney tubular cell injury and renal fibrosis^16, 17^. In the current study, we demonstrated a critical role of RTN1A in mediating ER stress and podocyte injury in DN. Expression of RTN1A in podocytes was confirmed here by co-staining with podocyte marker. In addition, a role of RTN1A in regulation of ER stress and apoptosis was demonstrated in cultured podocytes. These data suggest that RTN1A contributes to both glomerular and tubular cell injury in DN through regulation of ER stress. However, the exact role of RTN1A in podocyte injury in DN needs to be further confirmed *in vivo* using a podocyte-specific knockout approach.

Severe cellular insults in diabetes such as inflammation, hypoxia and oxidative stress may induce ER stress and subsequently contributes to podocyte damage^24, 25^. ER stress was extremely activated in podocyte of proteinuric rats with passive Heymann nephritis^26^, focal segmental or puromycin induced minimal-change nephrotic syndrome^27^, suggesting a relationship between proteinuria and ER stress. Persistent proteinuria is considered a strong predictor for the progression of renal disease^8^. It has been reported that proteinuria contributes to glomeruli dysfunction in DN^8^ and ER stress is one of the important mechanisms in albumin-induced podocyte injury^28^. Here, we confirmed that albumin can induce ER stress and apoptosis of podocytes and that this effect is inhibited by knockdown of RTN1A expression, suggesting RTN1A may mediate albumin-induced ER stress in podocytes. This is consistent with our recent *in vivo* studies showing that knockdown of RTN1A attenuated renal fibrosis in albumin overload nephropathy model^17^.

Over the past decade, there has been a considerable interest in developing compounds that modulate ER stress response. Chemical chaperones that improve ER folding capacity such as 4-phenylbutyric acid (4-PBA), TUDCA, and the ER chaperone ORP150, have been shown to reduce ER stress, restore glucose tolerance and improve insulin action and sensitivity^29–31^. TUDCA is an effective inhibitor of ER stress^31^. It is a hydrophilic bile acid that is normally produced endogenously in humans at very low levels^32^. Recent reports have shown that TUDCA can modulate ER function both *in vitro* and *in vivo* ^31, 33^ via stabilizing protein conformation, improving the folding capacity of the ER, facilitating the trafficking of mutant proteins and preventing advanced glycation end products induced apoptosis by blocking an ER stress-mediated apoptotic pathway in cultured mouse podocytes^34^. Here, we confirmed that TUDCA significantly attenuated proteinuria and kidney injury in db/db mice with Unx. TUDCA treatment also improved podocyte injury in diabetic mice. Interestingly, TUDCA reduced RTN1A expression in parallel to ER stress marker expression, further confirming a role of RTN1A in regulation of ER stress. This is further validated *in vitro* showing that RTN1A induced ER stress was inhibited by TUDCA treatment. These data further suggest a key role of RTN1A in mediating ER stress in DN.

How RTN1A mediates ER stress remains unclear. In our previous study, we confirmed a direct interaction between RTN1A and PERK in kidney cells and that this interaction is required for RTN1A-mediated activation of ER stress and apoptosis pathway^16^. PERK is known to be an important UPR sensor in the ER and is activated through phosphorylation under ER stress, while CHOP is a proapoptotic transcriptional factor that located downstream of PERK^35, 36^. We have shown that overexpression of RTN1A increases PERK phosphorylation leading to CHOP expression in kidney cells, whereas knockdown of RTN1A prevented tunicamycin or albumin induced phosphorylation of PERK and activation of CHOP^16^. It is known that CHOP deletion can protect renal tubular epithelial cells from apoptosis through inhibition or PERK/CHOP pathway^37^. *In vivo*, CHOP deficiency decreases renal cell apoptosis in murine models of unilateral ureteral obstruction (UUO) or renal ischemia/reperfusion injury^38, 39^. These data suggest a critical role of CHOP in kidney injury. Here, we found that knockdown of CHOP suppressed RTN1A expression and RTN1A-mediated ER stress and apoptosis in albumin-induced human podocytes, suggesting RTN1A expression is regulated by CHOP. These data together suggest a mechanism that RTN1A induces PERK phosphorylation to induce CHOP expression and CHOP, in turn, enhances RTN1A expression, forming a positive feedback manner to synergistically stimulate ER stress in podocytes.

In conclusion, we have shown that TUDCA, an ER stress inhibitor, improves diabetic kidney injury in a murine model with progressive DN. This is associated with significant attenuation of podocyte injury and suppression of ER stress markers in glomeruli. In addition, RTN1A expression, which was increased in diabetic mice, was also suppressed by TUDCA, confirming its role in ER stress. Furthermore, we confirmed that RTN1A mediates albumin-induced ER stress in cultured podocytes. Finally, our data suggest a positive feedback regulation between RTN1A and CHOP to enhance ER stress in podocytes and therefore inhibition of RTN1A could be a potential therapy to block ER stress-induced podocyte injury in DN. Overall, our data suggest a critical role of RTN1A and ER stress in podocyte injury in DN.

## Methods and Materials

### Animal models

Male diabetic db/db (C57BLKS/J-LepRdb/LepRdb) mice and their lean non-diabetic littermates db/m (C57BLKS/J-LepRdb/+) mice were purchased from the National Mode Animal Centre of Nanjing University (Nanjing, China) and housed under a constant 12-h light–dark cycle at a temperature between 21 °C and 23 °C and allowed free access to food and water in the SPF room. The animal experiment was approved by the Laboratory Animals Ethical Committee of Sixth People’s Hospital Affiliated to Shanghai Jiao Tong University. All methods were performed in accordance with the relevant guidelines and regulations. The db/db mice were subjected to uninephrectomy or sham surgery under anesthesia at the age of 8 weeks as described previously^40^. The db/m mice received sham surgery and served as the operation control. After 2 weeks of observation, db/db mice with one kidney were randomly divided into two groups. The operation group was intraperitoneally injected with TUDCA (250 mg/kg) twice daily or equal volumes of saline in control groups. During the experiment, the dosage was adjusted according to the body weight of the mice, the indicators were monitored regularly, and all mice were sacrificed at 16 weeks. All the experiments were repeated thrice.

### Urine albumin and creatinine

Timed (12-hour) urine collections were obtained from mice using metabolic cages. Urine protein was measured by an ELISA kit (Bethyl Laboratory, Houston, TX) for albumin, and urine creatinine levels were quantified using a QuantiChrom Creatinine Assay Kit (DICT-500; Bioassay Systems). Urine albumin excretion was expressed as the UACR. As for the reproducibility of this assay, the coefficients of variance (CV) was less than 3% when the same sample was measured three times consecutively.

### Measurement of serum creatinine (sCr)

Serum creatinine level was determined by use of a QuantiChrom creatinine assay kit (DICT-500; BioAssay Systems, Hayward, CA) according to the manufacturer’s instruction. The level of serum creatinine was expressed as milligrams per 100 ml (dl).

### Histology and morphometry

Kidneys embedded in either paraffin or frozen in OCT compound were sectioned to 4 μm thickness for light or fluorescence microscopy. Periodic acid–Schiff (PAS) and hematoxylin-eosin (HE) staining were performed for histological analyses. For transmission EM, kidney cortex samples fixed in 2.5% glutaradehyde were sectioned, and FP width and GBM thickness was performed using Image J (NIH) on digitized TEM images as previously described^41^.

### Immunohistochemistry and immunofluorescencestaining

Paraformaldehyde-fixed paraffin embedded kidney tissue sections were used in the study. Slides were subjected to heat-mediated antigen retrieval in citrate buffer before the addition of primary antibodies against RTN1A, GRP78, CHOP and p-PERK overnight at 4 °C respectively. Finally, 3,3′-diaminobenzidine tetrahydrochloride substrate was used to produce the color reaction. A double-immunolabeling technique was used for immunofluorescence staining. Immunostaining, image acquisition, and fluorescence intensity quantification were performed as previously described^42^. Mouse antibody to RTN1A and a rabbit antibody to synaptopodin were from Abcam (Cambridge, MA, USA). P57 antibody was from Santa Cruz Biotechnology (Santa Cruz, CA). The extent of kidney staining in mice kidneys was semi-quantitatively scored in a scale of 0–4 by two independent investigators (score 0: absence of specific staining; score 1: <25% area has specific staining for RTN1A; score 2: 25 to 50%; score 3: 50–75%; score 4 > 75%).

### Glomerular isolation

Glomeruli were isolated by infusing with a suspension of magnetic iron oxide, Fe_3_O_4_ (Sigma-Aldrich, St. Louis, MO, USA) according to the method described previously^43^.

### Cell culture and transfection

The conditionally immortalized human podocyte cell line was kindly provided by Dr. John Cijiang He (Icahn School of Medicine at Mount Sinai, New York, USA.), and the cells were cultured as previously described^16^. Podocytes were transiently transfected with RTN1A (QIAGEN, GmbH, Hilden, Germany) and pcDNA3.1(+) using ViaFect™ Transfection Reagent (Promega, Madison, WI, USA) according to the manufacturer’s protocols respectively. These cells were used for the following experiments after transfection for 48 h. The RTN1A-transfected cells were treated with or without TUDCA conditions. Podocytes were transiently transfected with shRNA-RTN1A and siRNA-CHOP using ViaFect^TM^ Transfection Reagent (Promega, Madison, WI, USA) according to the manufacturer’s protocols. For albumin overloading experiments, human podocytes were cultured with Endotoxin-free human serum albumin (Sigma-Aldrich, St. Louis, MO, USA).

### Apoptosis analysis

DeadEnd^TM^ Colometric TUNEL System from Promega (Promega, Madison, WI, USA) was used to detect apoptotic cells on formalin-fixed, paraffin-embedded kidney sections followed manufacturer’s instructions. Four-micrometer sections were then incubated with proteinase K (20 µg/ml in 10 mM Tris-Cl, pH 7.6, for 15 min at room temperature), blocked in 3% H_2_O_2_ for 10 min at 37 °C and treated with TUNEL reaction mixture. FITC Annexin V Apoptosis Detection Kit (BD Co Ltd) was used to detect the apoptosis rate of cultured podocytes according to the manufacturer’s protocols. The number of cells labeled with Annexin V-FITC and propidium iodide was quantified using the flow cytometer (Beckman Coulter, Beckman Coulter Inc, California, USA) and the data were analysed with CellQuest software (BD Biosciences).

### Real-time polymerase chain reaction

Total RNA was extracted from isolated glomeruli or cultured podocytes. RNA was reverse-transcribed using the Superscript III First-Strand Synthesis Super Mix (Invitrogen, Carlsbad, CA, USA). PCR was performed using SYBR Green Master Mix (Qiagen) with the Applied Biosystems step one plus Real-time PCR system (Applied Biosystems, Foster City, CA, USA). The homogeneity and the size of PCR amplicons were confirmed by melting curve analysis. Relative quantification of gene expression was carried out using the 2^−ΔΔCT^ method. Samples were executed in triplicates in separate tubes to permit quantification of the target gene normalized to GAPDH.

### Western analysis

Tissue or cells were lysed with RIPA buffer containing protease and phosphatase inhibitor cocktail. After quantifying protein concentration, lysates were subjected to western blot analysis using specific antibodies against p-PERK (Santa Cruz Biotechnology, Santa Cruz, CA), PERK, c-caspase3, CHOP, GAPDH, GRP78 (Cell Signaling Technology, MA, USA), RTN1A (Abcam, San Francisco, CA, USA) and FLAG (Sigma-Aldrich, St. Louis, MO). We repeated each Western blot analysis using protein from three different and separate experiments. The specific protein bands were scanned using Western Blotting Detection System (BIO-RAD).

### Statistical analysis

Data are shown as the mean ± SEM. ANOVA followed by Bonferroni correction was used to analyze means between more than two groups. And unpaired t test was used to analyze data when two groups were present. GraphPad Prism 5 software was used for statistical analyses. P values < 0.05 were considered statistically significant.

## Electronic supplementary material

Supplementary Information
