# Supplementary material for: Rtn1a-Mediated Endoplasmic Reticulum Stress in Podocyte Injury and Diabetic Nephropathy
Source: Sci Rep. 2017 Mar 23;7:323. doi: 10.1038/s41598-017-00305-6 (PMC5428279; doi:10.1038/s41598-017-00305-6)
Supplement: Supplementary file 1 — Supplementary Information [file 41598_2017_305_MOESM1_ESM.pdf]

RTN1A-MEDIATED ENDOPLASMIC RETICULUM STRESS IN PODOCYTE INJURY AND DIABETIC NEPHROPATHY

Ying Fan[1,+], Jing Zhang[1,+], Wenzhen Xiao[2], Kyung Lee[2], Zhengzhe Li[2], Jiejun Wen[1], Li He[1], Dingkun Gui[1], Rui Xue[1], Guihua Jian[1], Xiaohua Sheng[1], John Cijiang He[2, 3,*] and Niansong Wang[1,*]

[1]Department of Nephrology, Shanghai Jiao Tong University Affiliated Sixth People's Hospital, Shanghai, China; [2]Department of Medicine, Division of Nephrology, Icahn School of Medicine at Mount Sinai, NY United States; and [3]Renal Section, James J Peter Veterans Administration Medical Center, Bronx, NY, United States

[+]Ying Fan and Jing Zhang contributed equally to the study.

***Corresponding author:**

1. Niansong Wang, M.D.

Department of Nephrology

Shanghai Jiao Tong University Affiliated Sixth People's Hospital

600 Yishan Road, Shanghai, 200233, China

Tel: +86-021-64369181, Fax: +86-021-64701361

Email: wangniansong2012@163.com

2. John Cijiang He, M.D.

Division of Nephrology, Box 1243

Mount Sinai School of Medicine

One Gustave L Levy Place

New York, NY 10029

Tel: 212-659-1703, Fax: 212-987-0389

Email: cijiang.he@mssm.edu

Supplementary Table S1:

	db/m-sham-16wk	db/db-sham-16wk	db/db-sham-20wk	db/db-Unx-16wk
Body Weight (BW,g)	26.27±1.69	54.53±4.70*	55.00±6.04*	46.43±4.02*
Kidney Weight (KW,mg)	156.8±5.75	239.0±20.78*	222.6±13.63*	369.0±10.86*$^{\#\phi}$
KW/BW ratio (mg/g)	5.99±0.50	4.43±0.81	4.11±0.72*	7.98±0.56*$^{\#\phi}$
Blood glucose (mM)	8.0±1.2	27.6±1.9*	25.7±1.2*	27.3±4.2*
sCr (mg/dl)	0.25±0.09	0.43±0.11	0.62±0.13*	0.98±0.10*$^{\#\phi}$
UACR (ug/mg)	54.8±12.6	1074.1±171.5*	1473.9±181.9*	2011.1±130.3*$^{\#\phi}$

Supplementary Table S1. Characteristics of db/m and db/db mice. A graphic presentation of urinary albumin-to-creatinine ratio in db/m and db/db mice. Data are expressed as mean ± SEM. *P<0.05 compared with db/m-sham-16wk mice; *P<0.05 vs db/m-sham-16wk groups; $^{\#}$P<0.05 vs db/db-sham-16wk mice; $^{\phi}$P<0.05 vs db/db-sham-20wk mice (n=8 for each group).

Supplementary Table S2: Primer sequences

Species	Primers	Sequence
Human	H-RTN1A-QF1	TTGCCATGGAAACTGCATCC
	H-RTN1A-QR1	TGTAGAATCCTCCTGAGGTG
	H-GRP78-QF1	AACCGCTGAGGCTTATTTGG
	H-GRP78-QR1	TAGGCCAGCAATAGTTCCAG
	H-CHOP-QF1	AAGGCACTGAGCGTATCATG
	H-CHOP-QR1	TGCTTTCAGGTGTGGTGATG
Mouse	M-RTN1A-QF1	ATGGAAACTGCATCCACA
	M-RTN1A-QR1	AAAAGTATGCAGAGTCCTC
	M-GRP78-QF1	AGGCTAAGAGAGCCTTGTCT
	M-GRP78-QR1	TCCAACACTTTCTGGACAGG
	M-CHOP-QF1	TTCACCTTGGAGACGGTG
	M-CHOP-QR1	CGCAGGGTCAAGAGTAGTG
	M-bim-QF1	CCTACAGACAGAACCGCAAGC
	M-bim-QR1	CATTTGAGGGTGGTCTTCAGC
	M-bax-QF1	GGTTGCCCTCTTCTACTTTGC
	M-bax-QR1	GCCGCTCACGGAGGAAG
	M-bcl2-QF1	CTACCGTCGTGACTTCGCAG
	M-bcl2-QR1	CCCACCGAACTCAAAGAAGG
	M-WT1-QF1	ACAACGCCCATCCTCTGC
	M-WT1-QR1	TACAAGAGTCGGGGCTACTCC
	M-nephrin-QF1	CAAGAAGTCGCTCATCCTGAAC
	M-nephrin-QR1	CCAAACACACCAGCCTCACC
	M-Synaptopodin-QF1	TGACATCCAGCCCAACACC
	M-Synaptopodin-QR1	CTGGCCGTCCTGTTGACC

Supplementary Table S3: List of antibodies

Antibodies	Company	Cat No	Species	Notes
**RTN1A**	ABCAM	ab8957	Mouse	Monoclonal
**GRP78**	Cell Signaling	#3177	Rabbit	Monoclonal
**CHOP**	Cell Signaling	#2895	Mouse	Monoclonal
**Cleaved caspase-3**	Cell Signaling	#9664	Rabbit	Monoclonal
**PERK**	Cell Signaling	#3192	Rabbit	Monoclonal
**P-PERK**	SANTA CRUZ	sc-32577	Rabbit	Polyclonal
**Synaptopodin**	ABCAM	ab101883	Rabbit	Polyclonal
**P57**	SANTA CRUZ	sc-8298	Rabbit	Polyclonal

Supplementary Figure S1

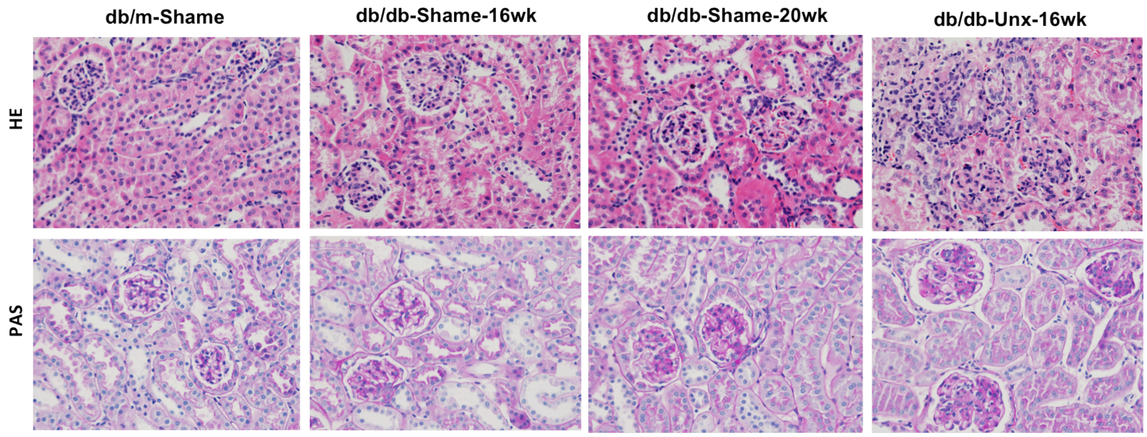

Supplementary Fig. S1: Unilateral nephrectomy accelerated kidney injury in db/db mice. db/db mice and db/m mice underwent unilateral nephrectomy (Unx) or sham operation (sham) at age of 8 weeks. Representative images of haematoxyline-eosin (HE) & periodic acid–Schiff (PAS) stained kidney sections of db/m and db/db mice at 16 weeks of age (original magnification, ×400).

Supplementary Figure S2: Full-length gels and blots in the main article

Fig. 2A

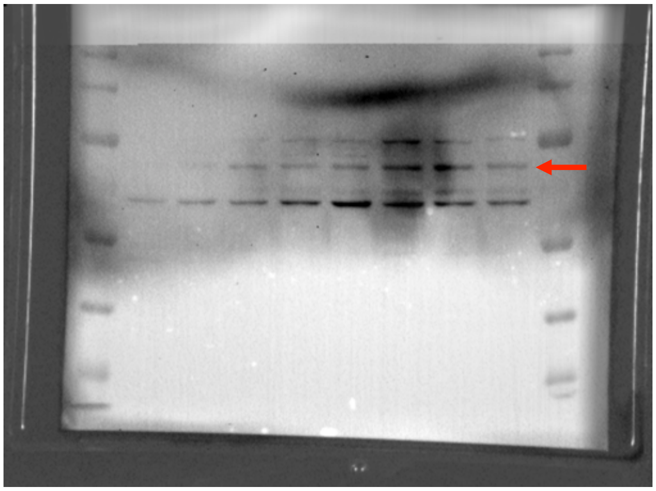

GRP78, MW 78KD

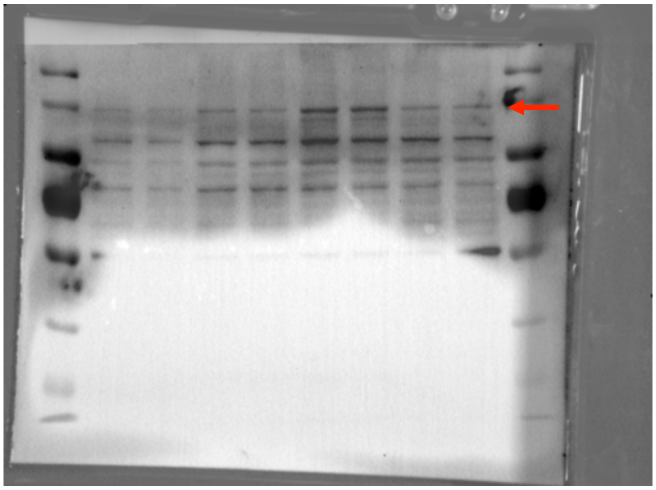

p-PERK, MW 125KD

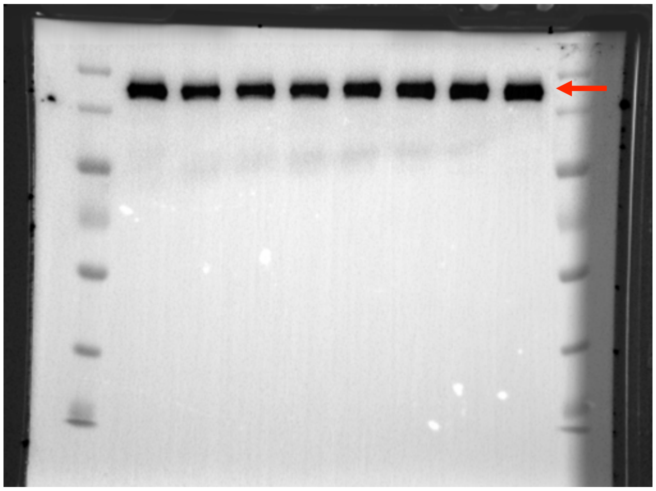

T-PERK, MW 140KD

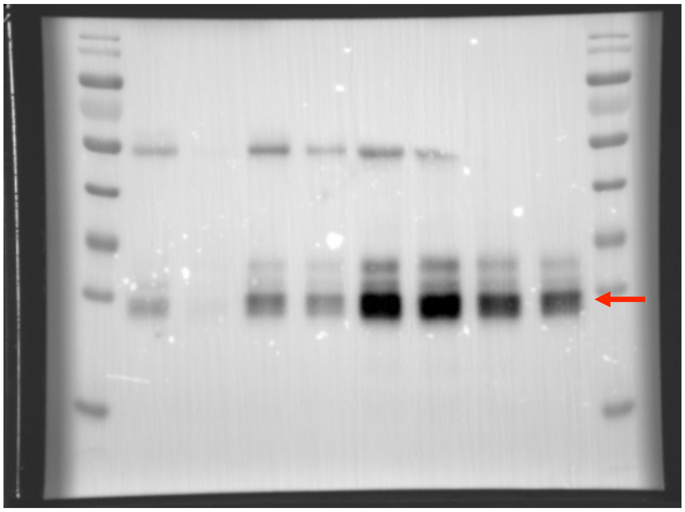

CHOP, MW 27KD

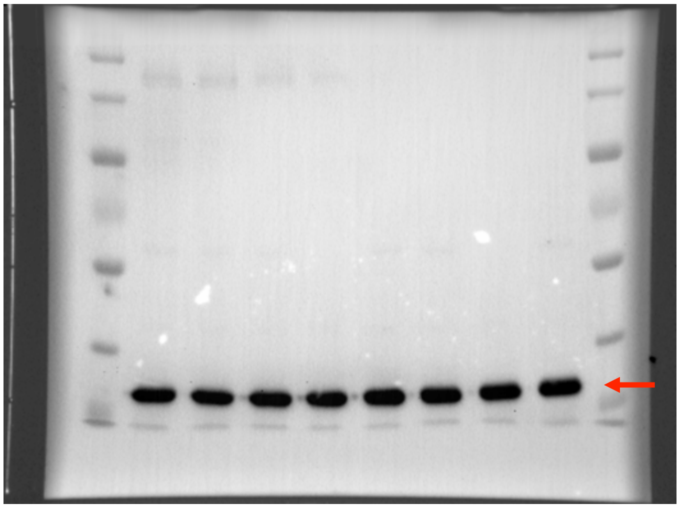

GAPDH, MW 36KD

Fig. 4A

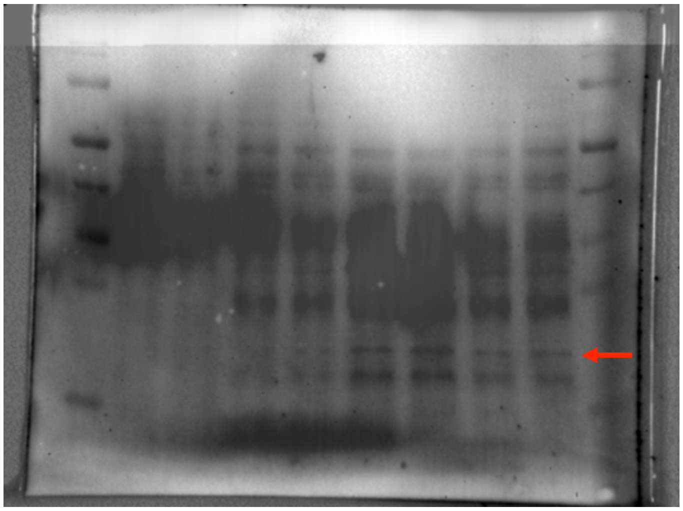

c-caspase3, MW 17,19KD

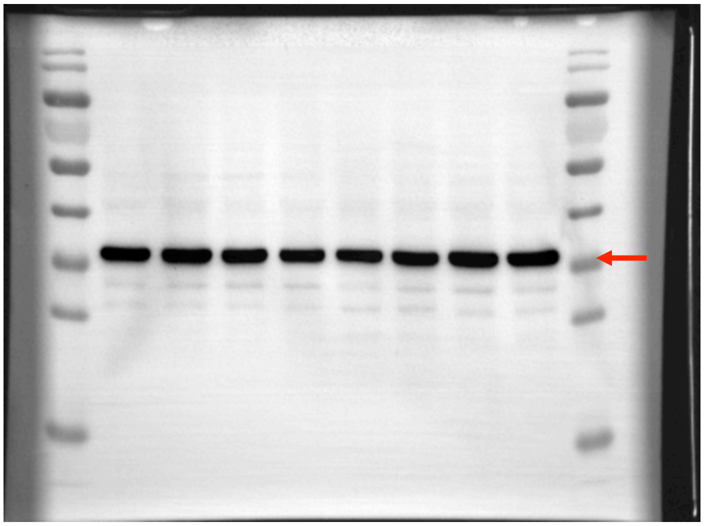

GAPDH, MW 36KD

Fig. 6A

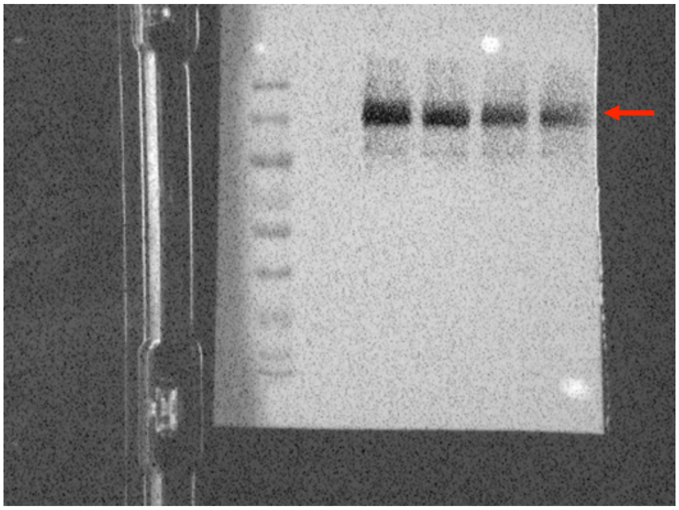

RTN1A, MW 135KD

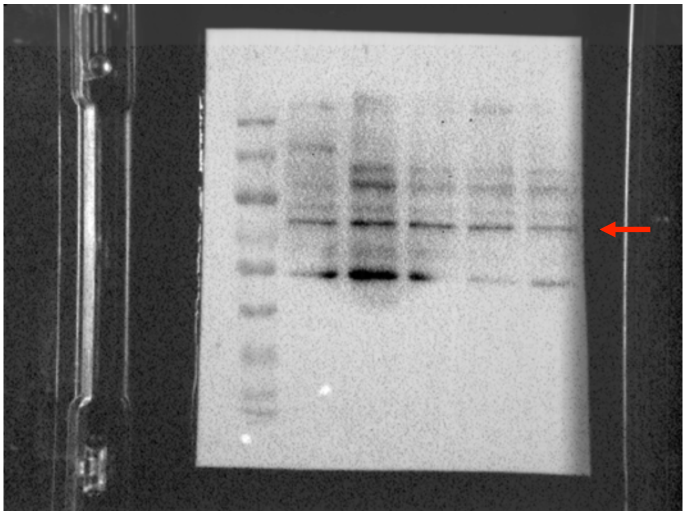

GRP78, MW 78KD

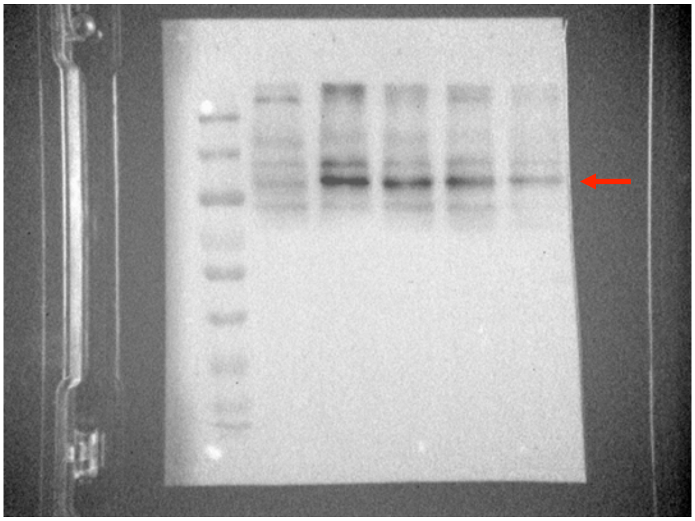

P-PERK, MW 125KD

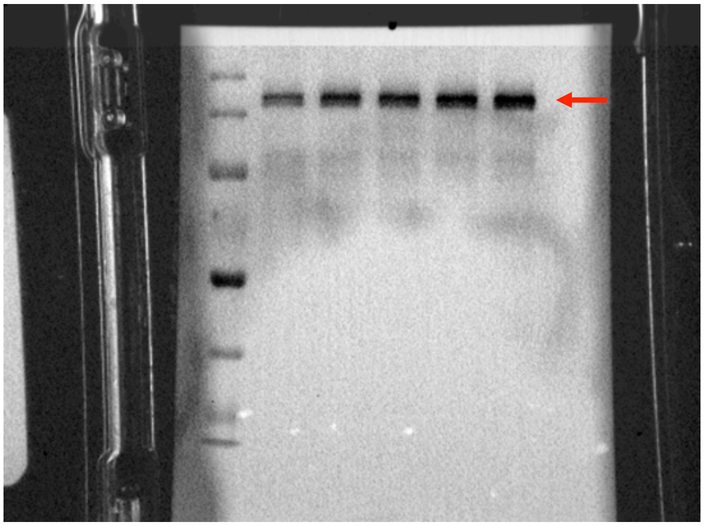

T-PERK, MW 140KD

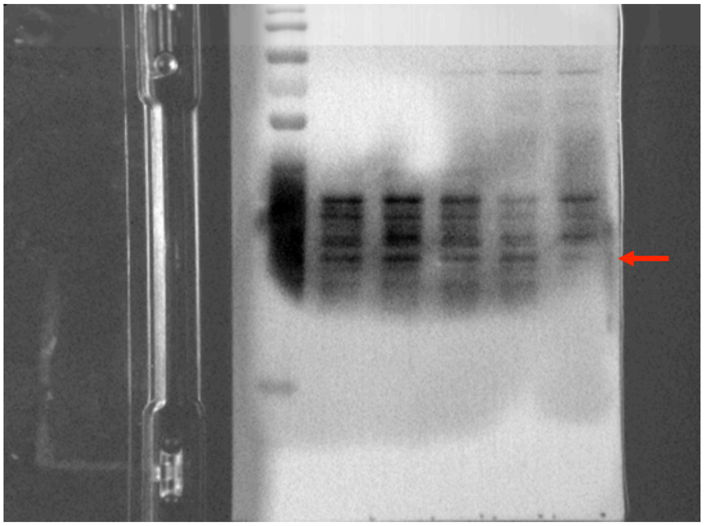

CHOP, MW 27KD

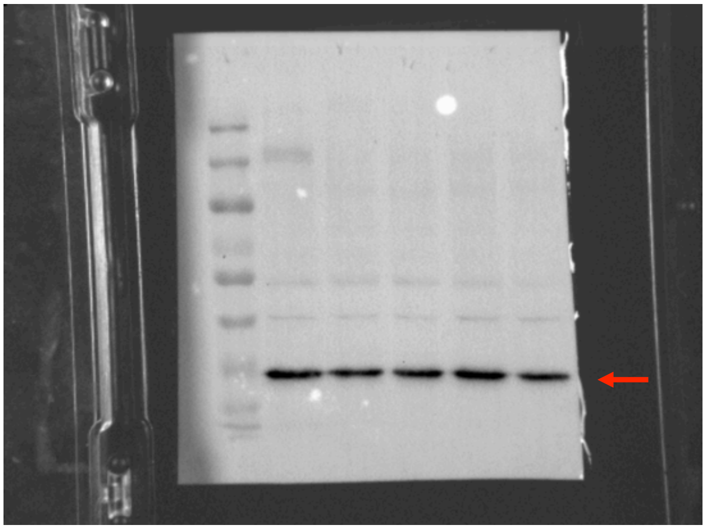

GAPDH, MW 36KD

Fig. 6D

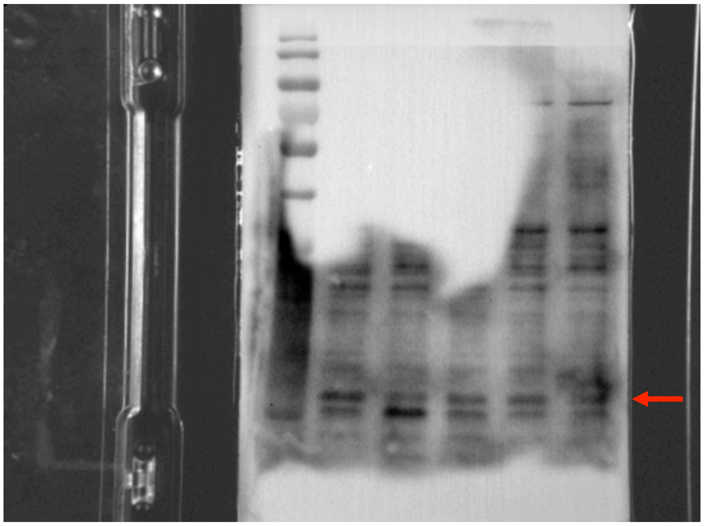

c-caspase3, MW 17,19KD

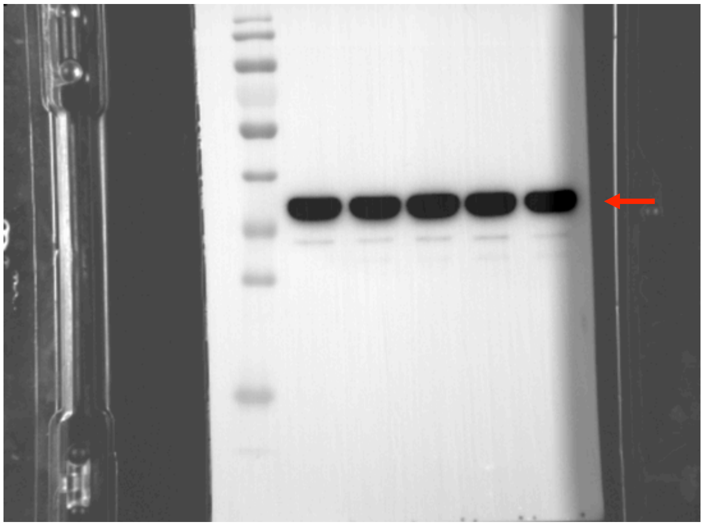

GAPDH, MW 36KD

Fig. 7A

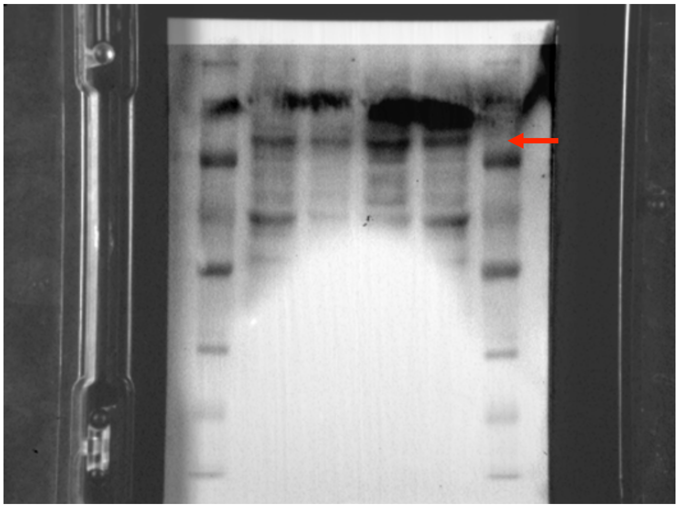

RTN1A, MW 135KD

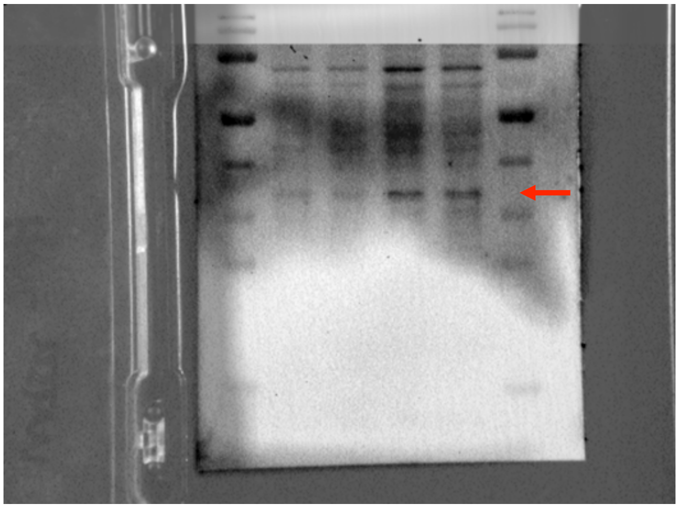

GRP78, MW 78KD

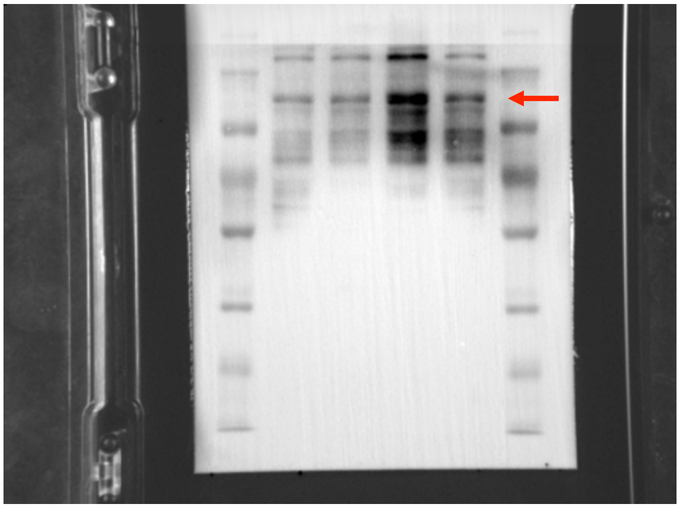

P-PERK, MW 125KD

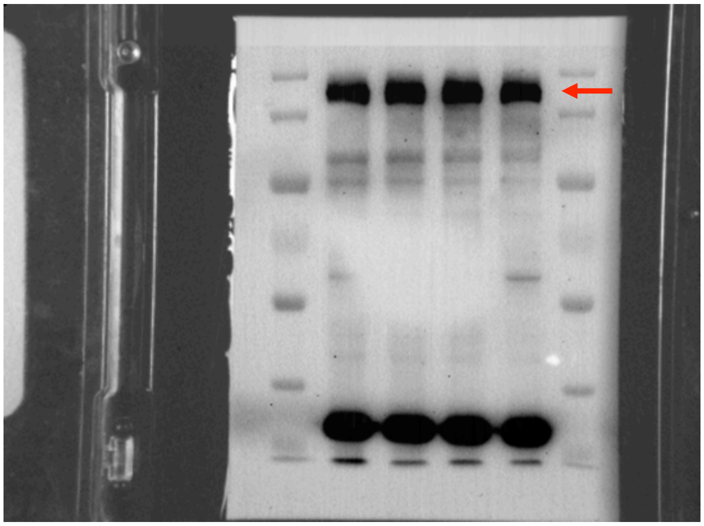

T-PERK, MW 140KD

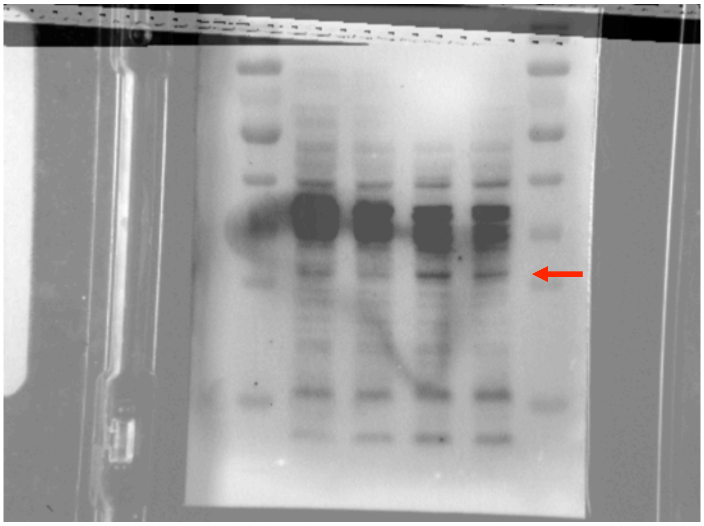

CHOP, MW 27KD

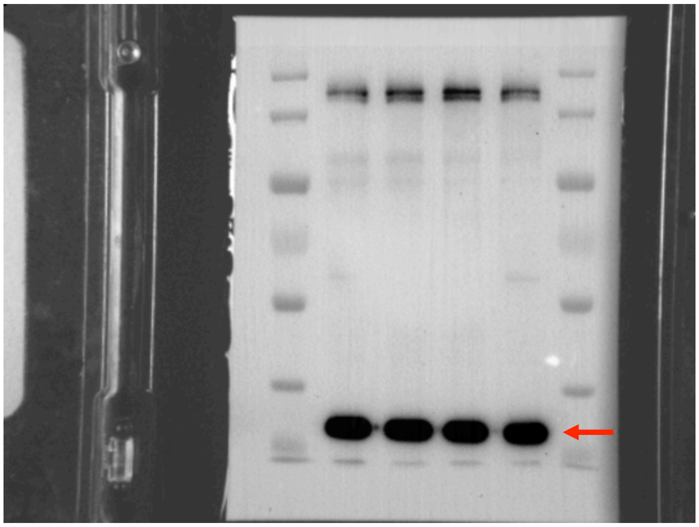

GAPDH, MW 36KD

Fig. 7D

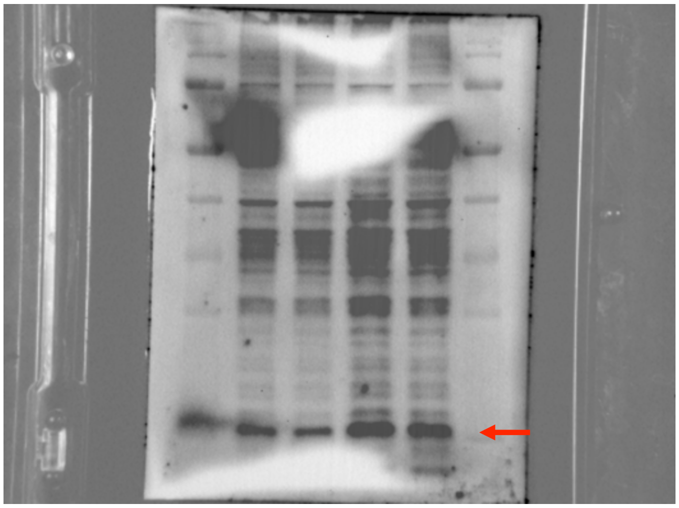

c-caspase3, MW 17,19KD

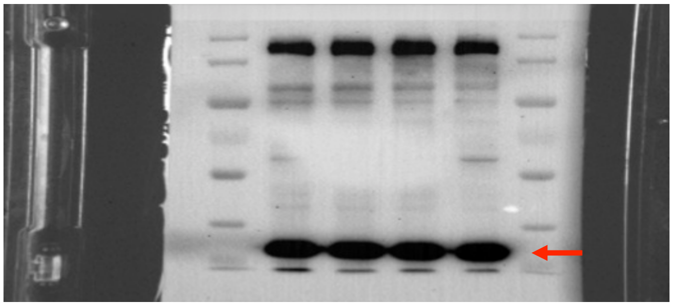

GAPDH, MW 36KD

Fig. 8A

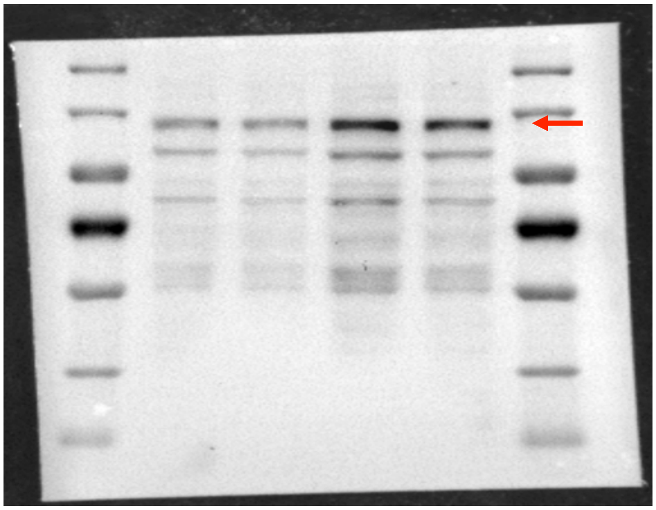

RTN1A, MW 135KD

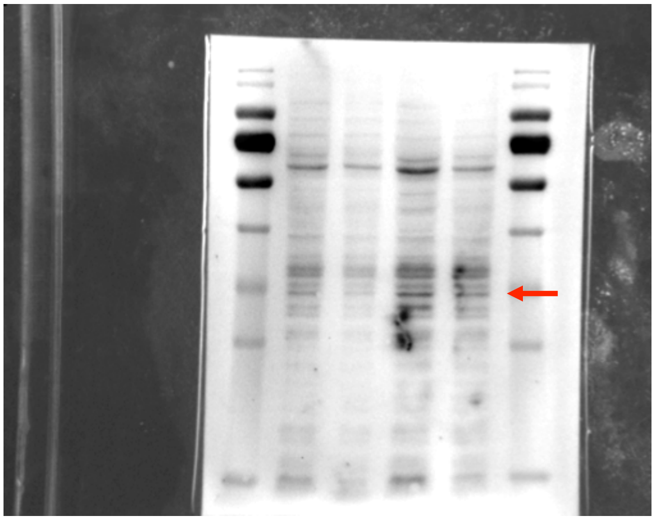

CHOP, MW 27KD

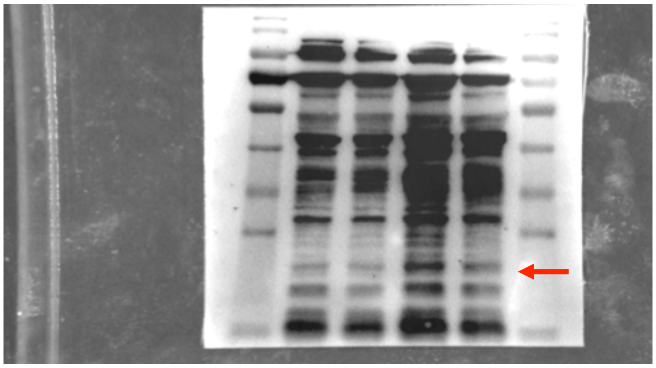

c-caspase3, MW 17,19KD

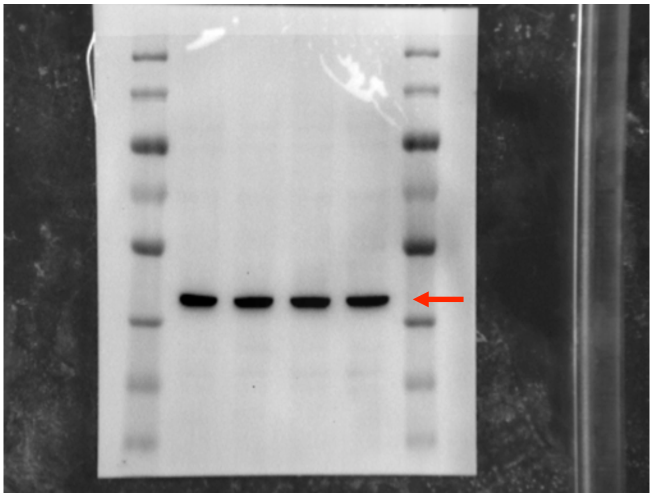

GAPDH, MW 36KD
